# Compliance status of tobacco control laws in the university setting of Bangladesh: an analytical study followed a mixed-method approach

**DOI:** 10.1136/bmjph-2023-000496

**Published:** 2024-05-02

**Authors:** Nasrin Akter, Bilkis Banu, Sujana Haque Chowdhury, Tahsin Tasneem Tabassum, Sarder Mahmud Hossain

**Affiliations:** 1 Northern University Bangladesh, Dhaka, Bangladesh; 2 Department of Public Health, Northern University Bangladesh, Dhaka, Bangladesh; 3 Department of Community Medicine, East West Medical College, Dhaka, Bangladesh

**Keywords:** Public Health, Education, Epidemiologic Methods, legislation and jurisprudence

## Abstract

**Background:**

Implementation of National Tobacco Control Laws (NTCLs) in university settings was found to be worse in Bangladesh.

**Objectives:**

This study aimed to depict the compliance status of tobacco control laws (TCLs) and the associated factors following the university setting approach in Bangladesh.

**Methods:**

This cross-sectional study was conducted with a mixed-method approach among the 385 students (quantitative method), 10 teachers and 10 administrative staff (qualitative method) of Northern University Bangladesh. Data were collected through mobile phone calling (quantitative) and in-depth interviews method (qualitative). Appropriate ethical issues were addressed. Logistic regression analysis was performed to find out the determinants. The study revealed non-satisfactory compliance with TCLs among 56.4% of study subjects which was strongly supported by the outcome of the qualitative approach. Predominant non-satisfactory compliance was found through the explorations of the conditions of buying and selling tobacco (78.4%), pasting no-smoking signage (3.2%), antitobacco advertisement within the university (34.8%) and specific university policy to implement NTCL. Non-satisfactory compliance was found significant among the younger aged (≤21 years: COR/p=2.74/0.01; 95% CI 1.27 to 5.92) from the first-year group (adjusted odds ratio (AOR)/p=2.28/0.02; 95% CI 1.15 to 4.49) who had moderate nicotine dependency (AOR/p=4.04/0.01; 95% CI 1.28 to 12.74), poor knowledge on TCLs (AOR/p=3.57/0.02; 95% CI 1.28 to 9.95) and the respondents who suggested family guidance (AOR/p=1.77/0.06; 95% CI 0.97 to 3.22) might be a sustainable way to minimise tobacco consumption in the university setting.

**Conclusions:**

The study revealed some crucial factors for the non-satisfactory compliance status towards TCLs in the university setting. This empirical outcome and evaluation strategy can guide to plan of future large-scale studies, which may lead to implementing effective intervention programmes focusing on the development of a tobacco-free environment in the university setting.

**WHAT IS ALREADY KNOWN ON THIS TOPIC**

- Previous and present reported studies have established that tobacco consumption initiations mostly happened by the influence of peer circles of educational institutions in Bangladesh, which facilitate tobacco availability among the students in an open secret manner by the student syndicates.

**WHAT THIS STUDY ADDS**

- This study will provide new insight into the compliance status with tobacco control laws (TCLs) in the university setting and the status of university policy regarding the implementation of TCLs. This study concluded by associating some sociodemographic and tobacco consumption-related determinants with non-satisfactory compliance with TCLs among university students.

**HOW THIS STUDY MIGHT AFFECT RESEARCH, PRACTICE OR POLICY**

- The outcome of this study might help to develop new approaches and strategies towards a tobacco-free environment in all universities of Bangladesh. In addition, this study will guide future large-scale nationwide studies to generate more scientific evidence for effective utilisation in successful implementation policies in educational settings.

## Background

 Worldwide, smoking is a major and expanding public health issue, with low-income and middle-income nations accounting for an excessive percentage of tobacco-related deaths.^1^ Evidence indicates prolonged smoking can cause various health issues, eventually leading to premature morbidity and mortality.^2 3^ Usually, university students are considered high-risk groups in terms of adopting risky behaviour, such as smoking and substance abuse. Alarmingly, the smoking habit among Bangladeshi students is gradually increasing.^4 5^

Initiation and continuation of smoking are believed to be multifactorial and vary according to age. Sometimes educational institutions and family structures play a role as an imposed burden on university students resulting in rising smoking habits among these particular groups of smokers^2^ and sometimes, the main reason for starting or continuing cigarette smoking is pleasure and fun among adults.^6^ Impending peer pressure can also be a significant contributing factor where some students smoke to indicate their transgression into adulthood—such behaviour is associated with different sociocultural groups on campus.^7^ Accessibility of cheap tobacco products, parental smoking and weak enforcement of laws play a role too in this ubiquitous habit of private university students. Among the original non-smoker university students, one-third have been seen to become regular smokers by demonstrating that by the time of their graduation. Once a student initiates smoking, he likely develops a regular smoking habit.^8^ The estimated cost of tobacco consumption by students constitutes 10% of their income which is diverted from the budgets allocated for nutrition, education and medical care.^9 10^ Therefore, smoker students are vulnerable to attracting a horde of illnesses that may result in significant afflictions.

Intending to curb this burning issue, the Government of Bangladesh implemented the Smoking and Tobacco Products Usage (Control) Act 2005. Also, it brought some amendments to the existing Smoking and Tobacco Control Amendment Act in 2013. Fine penalties for public smoking or compulsory printing of graphical health warnings on cigarette packets are some of the noteworthy laws enacted to date. For educational institutions, the laws reflect that educational institutions should not be marked off or designated as smoking areas, there should be arrangements to display a warning notice containing ‘refrain from smoking, it is a punishable offence’ in the directed format at the entrance and one or more places inside; not to allow consumption or sale of tobacco within 100 yards of the educational institution premises; prohibit Tobacco Advertising, Promotion and Sponsorship in educational institutions, there should be antitobacco advertisements in educational institutions.^11^

Previous academic and scientific studies have provided evidence regarding the prevalence of tobacco use as well as the underlying causes of tobacco initiation in rural communities and among university or medical students. This research, however, did not investigate the effects of TCLs compliance in university settings among academic personnel and students in Bangladesh but rather showed the distribution of tobacco consumption especially among private university students.^1 5 7 8^ Much research has yet to be conducted regarding the causes of tobacco smoking and compliance with tobacco control laws (TCL) among private university students, including the respective authorities in a university. In the demographic aspect, Bangladesh has a higher proportion of youths than other age groups. This youth population of students with diverse socioeconomic status has occupied private universities in a considerable percentage to their convenience. Smoking is highly addictive and, as a result, can jeopardise the country’s future by crippling the next generation.^12^ It is high time to take action to establish a society free from tobacco use, starting with legislation that targets particularly our youth.

Therefore, this study aimed to portray the compliance status of TCLs in all tires of university settings, including the implementation policy. In line with that, an astute exploration of the underlying factors was also intended here, which were responsible for the non-compliance status of the university students. Moreover, this study was also considered to reveal some significant barriers and ways to minimise tobacco consumption in the university area. The practical outcomes attained from this study can prove to achieve a glorious feat in applying successful strategies to establish the universities as the complete tobacco-free zone in Bangladesh.

## Methods

### Study design and setting

This descriptive type of cross-sectional study was carried out based on a mixed method approach, that is, quantitative and qualitative designs. Data were collected from January to June 2021 to depict the compliance status of TCLs of Bangladesh following the university setting approach. Considering the variations of academic settings, that is, students, teachers, staff and university policies, the study was conducted in Dhaka city of Dhaka district. Moreover, the sociocultural norms and views are nearly similar in all the private and public universities and regulated by the University Grants Commission under the same rules and regulations. From that point of view, this study was conducted among the students, teachers and staff of the first leading digital private university, that is, Northern University Bangladesh (NUB).

### Study participants, sample size, sampling and public involvement

A total of 385 respondents were randomly enrolled in the quantitative part of this study who were undergraduate students and both male and female groups from the selected private university, that is, NUB. Study participants were recruited from all enrolled students in NUB’s Spring 2021 semester (January–April 2021). The potential standard sample size of 385 was calculated by using the formula ‘n= ‘Z2pq/d2’ where Z (standard normal deviate) was considered as 1.96; p (compliance with the smoke-free law in educational institutions)^13^ was considered as 65% and margin of error was considered as 0.05. With a minimum calculated sample of 350, an additional 10% of 350, that is, 35 samples was added as a cushion to account for non-response and questionnaire error factors, and the final samples were 385. Study subjects were selected by systematic random sampling following inclusion criteria and 385 students were chosen from the university-provided list of 2131 students studying in nine different university departments. Moreover, qualitative information was collected from 10 teachers and 10 administrative staff (involved with policy-making and implementation) of the university to obtain an insight snapshot according to the variables. There was no public involvement in the activities of this study, such as defining research questions and outcome measures, providing input into study design and conduct, dissemination of results, etc.

### Data collection

Quantitative data were gathered by pretested, semistructured and anonymous paper-based questionnaires through a telephonic conversation system. Due to the spread of the COVID-19 pandemic and the lockdown policy enforced within the country, a physical questionnaire was not feasible. Thus, respondents were accessed through a phone call system. Each respondent took only 10–15 min for the quantitative survey and 30–40 min for the qualitative survey. The phone call-based survey was administered in the local Bengali language with the utmost support of the university authority. In addition, qualitative information was collected by face-to-face in-depth interviews (IDIs). A total of 10 IDIs were conducted among the teachers, and 10 IDIs were conducted among the university’s administrative staff to explore the contributing factors and ascertain the unknown factors in detail. Interviews were taped and transcribed verbatim as immediately as possible after finishing each event.

### Questionnaire design

The quantitative and qualitative questionnaires were prevalidated by two independent reviewers and pretested among 5% of the respondents. The responses from the pretest were used to improve the quality of the questionnaire. The questionnaire comprised two pivotal segments: (a) independent variables and (b) dependent variables. Independent variables included (1) sociodemographic characteristics (age, religion, level of education of the respondents, parental education, parental occupation, monthly family income); (2) tobacco consumption pattern (tobacco consumption status, types of consuming tobacco, tobacco initiation age, duration of tobacco consumption, level identification by Fagerstrom Test for Nicotine Dependency (FTND), passive smoking status, family member smoking history); (3) knowledge regarding health hazards of tobacco intake and TCLs (knowledge on hazards of tobacco consumption, knowledge on TCLs); (4) barriers to minimise tobacco consumption (negative self-willingness, peer group influence, social media influence, insufficient knowledge on TCLs) and (5) way outs to minimise tobacco consumption (self-influence, friend’s influence, family guidance, teacher’s support, laws implementation by the University) and dependent variables of this study included compliance status on TCLs in the university campus (smoking in the campus, prohibit by friends, teacher’s advice, antitobacco advertisement, selling/ buying single stick tobacco and by minors, availability of smoking zone, ‘no Smoking’ sign in the campus, cafeteria and the university bus). A topic guide was prepared to conduct the qualitative interview. The topic guide’s components were based on this study’s objectives.

### Patient and public involvement

No patients or members of the general public were involved in the planning, execution, reporting or distribution of our study.

### Data analysis

Data quality was checked and qualitative data were analysed by content analysis and a constant comparative method. SPSS software (V.21) was used to analyse the quantitative data. Study characteristics were subjected to descriptive statistics (frequency and proportions) to summarise the obtained data. To categorise the data on age and monthly income, the cut-off value was decided according to previous relevant published articles.^14^ FTND contains six items that evaluate the quantity of cigarette consumption, the compulsion to use and dependence. In the scoring of FTND, yes/no items are scored from 0 to 1 and multiple-choice items are scored from 0 to 3. The items are summed to yield a total score of 0–10. The higher the total Fagerström score, the more intense is the patient’s physical dependence on nicotine.^15^ A scoring system was developed to categorise the participant’s knowledge (hazards of tobacco consumption and TCLs) and compliance. The score calculation included all the components related to knowledge and compliance. Only the correct answers to each question were listed. Each correct response was assigned a score of 1; each incorrect response was assigned a score of 0. The total score was summed up on the basis of corrected responses of individual answers of the question. Afterward, the total score was converted into percentages and classified into categories. Poor knowledge corresponded to a score of (<40%) and good knowledge corresponded to a score of (>40%).^16^ The level of compliance was classified into satisfactory (score considered as ≥70) and non-satisfactory (score considered as <70) status.^17^

A χ^2^ test was performed to observe the association between the compliance status of TCLs and the background characteristics of the university students. A multivariable logistic regression analysis was performed, followed by a modelling procedure considering the backward elimination process, including prespecified confounders followed by the independent variables. ORs with 95% CIs concerning compliance with TCLs were calculated for the specified exposures.

## Results

### Student’s characteristics: (quantitative approach)

For the quantitative approach, a total of 384 respondents were included in this study. Table 1 reveals that among the respondents, 59% (n=227/385) belonged to the age group of 22–24 years, and the highest (32.5%) group of students were from second year in their academic profile. The study also revealed that many subjects (32.7%, n=126/385) were tobacco smokers.

**Table 1:** Characteristics of the respondents (students) associated with compliance status in the university setting (n=385)

Characteristics	Compliance status			
	Number of participants, n (%)	Satisfactory, n (%)	Non-satisfactory, n (%)	P value (≤0.05)
Age (in years)				
≤21	51 (13.2)	11 (21.6)	40 (78.4)	0.01*
22–24	227 (59.0)	111 (48.9)	116 (51.1)	
>24	107 (27.8)	46 (43)	61 (57)	
Education				
First year	91 (23.6)	22 (24.2)	69 (75.8)	<0.01*
Second year	125 (32.5)	64 (51.2)	61 (48.8)	
Third year	91 (23.6)	50 (54.9)	41 (45.1)	
Fourth year	78 (20.3)	32 (41)	46 (59)	
Religion				
Muslim	331 (86.0)	136 (41.1)	195 (58.9)	<0.01*
Non-Muslim	54 (14)	32 (59.3)	22 (40.7)	
Tobacco initiation age (in years)				
09–19	82 (65.1)	28 (34.1)	54 (65.9)	<0.01*
20–27	44 (34.9)	26 (59.1)	18 (40.9)	
FTND Level				
Moderate	21 (5.5)	4 (19)	17 (81)	0.05*
High	15 (3.9)	8 (53.3)	7 (46.7)	
Low	349 (90.6)	156 (44.7)	193 (55.3)	
Knowledge on TCLs				
Good	20 (5.2)	14 (70)	6 (30)	0.02*
Poor	365 (94.8)	154 (42.2)	211 (57.8)	
Passive smoking status				
Yes	171 (44.4)	61 (35.7)	110 (64.3)	<0.01*
No	214 (55.6)	107 (50)	107 (50)	
Family member smoking history				
Yes	75 (19.5)	23 (30.7)	52 (69.3)	<0.01*
No	310 (80.5)	145 (46.8)	165 (53.2)	
Influence from peer group (barrier to minimise tobacco consumption)				
Yes	258 (67.0)	122 (47.3)	136 (52.7)	0.04*
No	127 (33.0)	46 (36.2)	81 (63.8)	
Family guidance (way outs to minimise tobacco consumption)				
No	317 (82.3)	147 (46.4)	170 (53.6)	0.02*
Yes	68 (17.7)	21 (30.9)	47 (69.1)	
Teacher’s support (way outs to minimise tobacco consumption)				
No	137 (35.6)	50 (36.5)	87 (63.5)	0.04*
Yes	248 (64.4)	118 (47.6)	130 (52.4)	

Data are presented as frequency (n), percentage (%).

* Statistical significance at p≤0.05. χ2 test was used to observe the association.

FTNDFagerstrom Test for Nicotine DependenceTCLsTobacco Control Laws

### Compliance status on TCLs in the university setting responded to the students: (quantitative approach)

**Figure 1:**
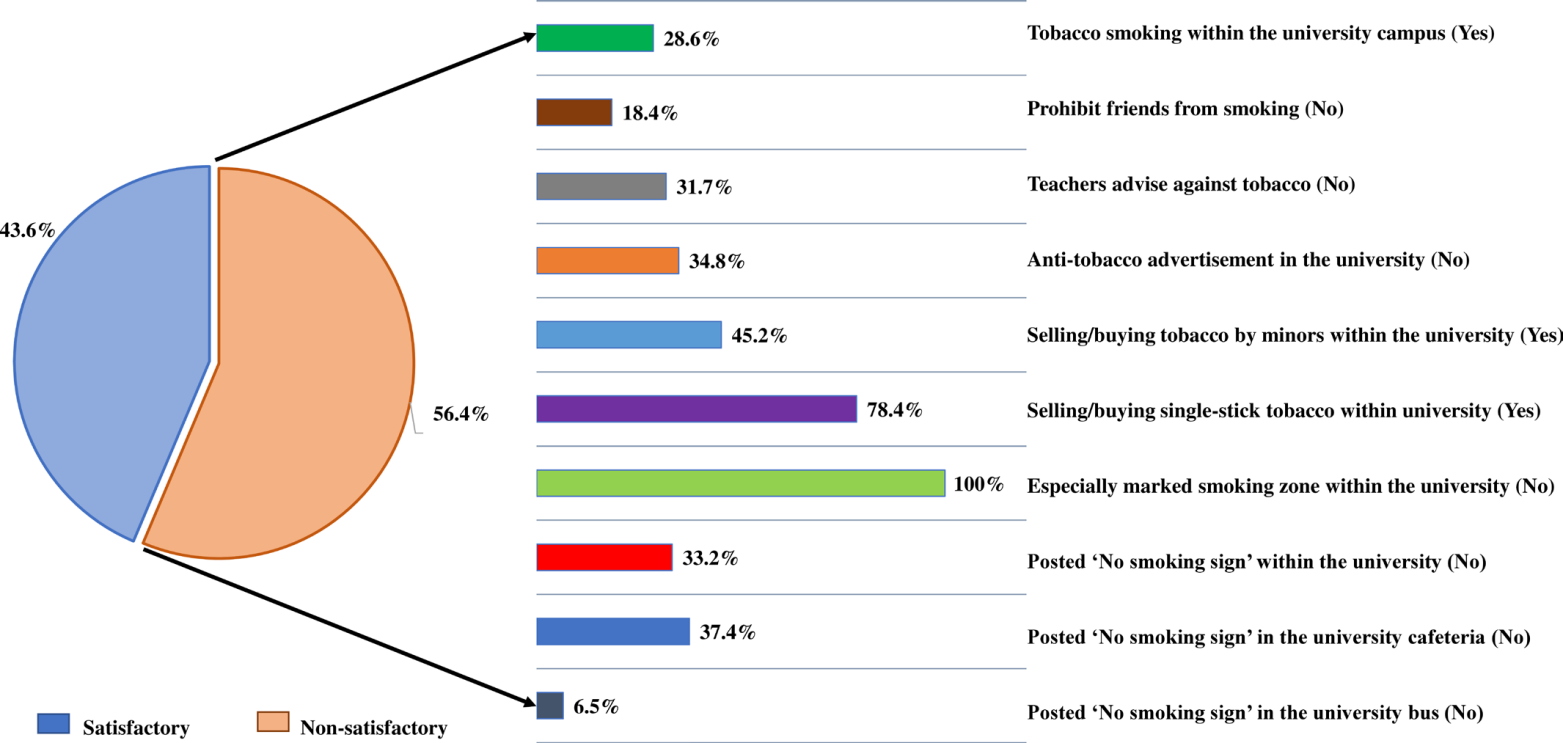
This is the compliance status of TCLs in the university setting (n=385). TCLs, tobacco control laws.

The overall compliance rate to TCLs within the university setting was found to be satisfactory among 43.6% and non-satisfactory among 56.4% of study subjects in figure 1. The compliance level was scored considering the adherence to basic regulations or provisions of the National Tobacco Control Laws (NTCLs) within the university. It was observed that complete (100%, n=385) non-adherence was reported on the ‘presence of marked smoking zone within the university area’. Furthermore, the regulation regarding buying/selling tobacco products shows that 78.4% (n=301/385) study subjects witnessed buying and selling single stick tobacco within the university campus, and 45.2% (n=173/385) subjects reported the non-adherence of the university to the regulation ‘buying/selling tobacco by the minors’ which should be prohibited within the university area. Only pasting a sign-board of ‘no smoking’ is a famous and effortless provision in the law. Unfortunately, findings relating to pasting the ‘no smoking sign’ were also disappointing. Likewise, 33.2% (n=123) and 37.4% (n=143) of study respondents did not find any ‘no smoking sign’ stickers within the university campus and university cafeteria, respectively, which is the outcome of the reluctant behaviour of higher authorities towards TCLs. In addition, instead of declaring ‘no smoking’ in public transport by NTCL, 6.5% of university students still didn’t notice the no smoking signage on university buses. Compliance level on TCLs related to teachers’ advice shows that 31.7% (n=121/385) of teachers do not advise their students to avoid tobacco. On the other hand, NTCLs remarked that a university area is a public place where smoking is completely prohibited. However, it is alarming to observe that 28.6% (n=110/385) of students agreed to smoke tobacco within their university premises.

### Compliance status on TCLs in the university setting responded to the teachers and administrative staff: (qualitative approach)

The qualitative approach of this survey also revealed a poor compliance status on TCLs in the university setting. Responses were taken from 10 faculty members and 10 administrative staff through IDIs. Here, 10 components (student’s consumption status within the university, teacher’s advice against tobacco, antitobacco advertisement, declaration of no smoke zone, no smoking signage, university policy on TCLs, implementation of fine penalty, inhibit sponsorship from the tobacco industry, antitobacco awareness programme, any action taken against tobacco) were considered to analyse the compliance status of the university as well as the individual respondents. Non-compliance behaviour was reported by all the teachers and administrative staff regarding tobacco consumption status among the university students, antitobacco advertisements, declaration of no smoking zone, no smoking signage, university policy on TCLs and implementation of fine penalty. To explain the smoking status among university students, participants mentioned that the majority of the students consume tobacco, mostly senior male students from the law, business and IT departments.

…students do not smoke in first year …Then …in third and fourth year they become the consumers by the influence of seniors in tea stalls. [I-T-1] (Teachers)

Both groups agreed to inhibit sponsorship from the tobacco industry as good compliance with TCLs. In addition, mostly good compliance was found regarding the pasting of ‘no smoking’ signage in the university, although the posters were not pasted according to the government standard. However, very few teachers and administrative staff were involved in antitobacco awareness programmes and took action against tobacco incidence. For instance, in two or three cases of teachers and admins, the study revealed that they took several actions against tobacco consumption like reducing scholarships and dropping the running semesters instead of a fine penalty. In some cases, reluctance from the compliance on TCLs was found due to less awareness of tobacco consumption, especially among the cases that led them not to advise students against tobacco. Sometimes, teachers and admins were smokers also, which is why they did not feel the importance of being aware of students against tobacco consumption.

To ask for the barriers to minimising tobacco in the university setting, responses found implementation-related barriers (no existing policies, no monitoring cells, absence of no-smoking signs (standard), lack of antitobacco advertisements), cultural barriers (peer pressure, a perceived sign of smartness, smokers get importance in a peer group), awareness related barriers (unaware about TCLs, the reluctance of complying law), lack of counselling/advice from teachers and guardians, availability of tobacco products and tobacco industry influence.

To explore the way outs, responses from the subjects revealed raising awareness, moral boosting, the establishment of the university as a non-smoking zone, proper implementation of NTCLs, showing the live examples of sufferers of tobacco, preaching the health hazards among the students, controlling social media against tobacco, teacher’s awareness.

The University should have anti-tobacco cells. Where representatives will be faculties, administrative staff, and nonsmoker students. There should be another monitoring system through which the university can monitor the all activities of the anti-tobacco cell, thus, everyone will do the work effectively. [I-T-10] (Teacher)

We have to show the sufferers of smoking such as give examples of patients of lung cancers and their families. We should also avoid smoking due to peer pressure. Overall preaching about the health hazards can bring about changes. [I-A-10] (Administrative staff)

### Student’s characteristics associated with compliance status in the university setting: (quantitative approach)

Students are undoubtedly considered the ultimate fighters in society against tobacco consumption. However, unfortunately, this study shows that students from the younger age group (≤21 years: 78.4%, n=40/51) were found to have significant (p=0.01) non-satisfactory compliance status compared with other age groups. It was surprisingly observed that students from first year reported a maximum (75.8%, n=69/91) non-satisfactory compliance level (p<0.01) on TCLs which may be as a result of less exposure to academic platforms and tobacco control activities. In addition, as prime factors which had an effective influence on non-satisfactory compliance; the study revealed the age of tobacco initiation (p<0.01) and nicotine dependency (p=0.05) of students (measured by FTND as statistically significant. Students who initiated tobacco consumption at a very early age of 9–19 (65.9%, n=54/385) with moderate levels of nicotine dependency (81%, n=17/385) were significantly associated with non-satisfactory compliance status to TCLs. Furthermore, considering the passive smoking status of tobacco, it was found that family history of smoking (69.3%, n=52/385) and passive smoking (64.3%, n=110/385) act as significant (p<0.01) influencing factors for non-satisfactory compliance status among the students. Moving towards the knowledge of TCLs among the students, the result shows that respondents with poor knowledge (94.8%, n=365/384) had poor compliance (57.8%, n=211/384) with TCLs. Finally, concerning barriers to minimising tobacco consumption in the university setting, non-satisfactory compliance on TCLs was found more significant among the subjects who did not agree with the barrier ‘influence from peer group’ (63.8%, n=81/127) compared with others. It might be the reflection of the respondent’s egoistic behaviour. Furthermore, significant non-satisfactory compliance was found among those groups who suggested family guidance (69.1%, n=47/68) and teacher’s support (63.5%, n=87/137) can be an effective way to minimise tobacco consumption in the university setting (table 1).

### Predictors for the non-satisfactory compliance towards TCLs among the students in a university setting: (quantitative approach)

Regression analysis of the study revealed significant predictors determining the non-satisfactory compliance status of TCLs within the university setting. Students from the younger age group (≤21 years: crude odds ratio (COR)/p=2.74/0.01; 95% CI 1.27 to 5.92) who were Muslim (COR/p=2.09/0.01; 95% CI 1.16 to 3.75) found to have significantly higher odds for non-satisfactory compliance status on TCLs. It was also observed that students enrolled in first year (COR/p=2.18/0.02; 95% CI 1.13 to 4.22) were more vulnerable to show less compliance with TCLs in compression to the counter groups. In addition, students with family history of smoking (COR/p=1.99/0.01; 95% CI 1.16 to 3.41) who had continuous passive exposure of smoking (COR/p=1.80/0.01; 95% CI 1.19 to 2.72) and had an early initiation of tobacco smoking (9–19 years: COR/p=2.79/0.03; 95% CI 1.13 to 10.41) were observed to have significant non-satisfactory compliance status compared with the counters. To explore the barriers, it was found that respondents who didn’t consider influence from peer group as a barrier (COR/p=1.58/0.04; 95% CI 1.02 to 2.44) had significantly less compliance with TCLs. Moreover, from the suggested way outs to minimise tobacco consumption in the university setting, significant predictors for poor compliance were identified as family guidance (COR/p=1.94/0.02; 95% CI 1.11 to 3.39) and teacher’s support (COR/p=1.58/0.04; 95% CI 1.03 to 2.42) with significant nearly two times higher odds compared with others.

After adjustment of modelling and eliminating the confounders in a backward manner, final predictors were identified which triggered the status of non-satisfactory compliance with TCLs among the university students. Junior students from the first year group (adjusted odds ratio (AOR)/p=2.28/0.02; 95% CI 1.15 to 4.49) who had moderate nicotine dependency (AOR/p=4.04/0.01; 95% CI 1.28 to 12.74) and poor knowledge on TCLs (AOR/p=3.57/0.02; 95% CI 1.28 to 9.95) had significant non-satisfactory compliance with TCLs compared with the counters. In addition, as a barrier to minimising tobacco, it was observed that, reluctant respondents who did not mention their peer group as a barrier (AOR/p=2.05/0.01; 95% CI 1.28 to 3.28) were found to have significantly higher odds for their non-satisfactory compliance with TCLs. Furthermore, to explore the predictors from way outs to minimise tobacco consumption, significant poor compliance status was found among the respondents who suggested family guidance (AOR/p=1.77/0.06; 95% CI 0.97 to 3.22) as a sustainable way out to control tobacco consumption among the university students (table 2).

**Table 2:** Predictors associated with non-satisfactory compliance on TCLs among the university students (n=385)

Characteristics	Compliance status			
	Non-satisfactory versus satisfactory			
	COR (95% CI)	P value	AOR (95% CI)	P value
Age group (in years)				
≤21	2.74 (1.27 to 5.92)	0.01*	─	─
22–24	0.79 (0.49 to 1.25)	0.79	─	─
>24	Reference			
Education				
First year	2.18 (1.13 to 4.22)	0.02*	2.28 (1.15 to 4.49)	0.02*
Second year	0.66 (0.37 to 1.17)	0.16	0.60 (0.33 to 1.09)	0.09
Third year	0.57 (0.31 to 1.05)	0.07	0.55 (0.29 to 1.04)	0.07
Fourth year	Reference			
Religion				
Muslim	2.09 (1.16 to 3.75)	0.01*	─	─
Non-Muslim	Reference			
Tobacco initiation age (in years)				
09–19	2.79 (1.31 to 10.41)	0.03*	─	─
20–27	Reference			
FTND Level				
Moderate	3.4 (1.13 to 10.41)	0.03*	4.04 (1.28 to 12.74)	0.01*
High	0.71 (0.25 to 1.99)	0.51	0.70 (0.24 to 2.05)	0.52
Low	Reference			
Knowledge on TCLs				
Good	Reference			
Poor	3.19 (1.20 to 8.51)	0.02*	3.57 (1.28 to 9.95)	0.02*
Passive smoking status				
Yes	1.80 (1.19 to 2.72)	0.01*	─	─
No	Reference			
Family member smoking history				
Yes	1.99 (1.16 to 3.41)	0.01*	─	─
No	Reference			
Influence from peer group (barrier to minimise tobacco consumption)				
Yes	Reference			
No	1.58 (1.02 to 2.44)	0.04*	2.05 (1.28 to 3.28)	0.01*
Family guidance (way outs to minimise tobacco consumption)				
Yes	1.94 (1.11 to 3.39)	0.02*	1.77 (0.97 to 3.22)	0.06*
No	Reference			
Teacher’s support (way outs to minimise tobacco consumption)				
Yes	1.58 (1.03 to 2.42)	0.04*	─	─
No	Reference			

Logistic regression analysis was used to identify the predictors.

* Statistical significance at p≤0.05; reference category was considered for compliance status on TCLs in the university setting as satisfactory compliance level.

AORadjusted odds ratioCORcrude odds ratioFTNDFagerstrom test for nicotine dependenceTCLstobacco control laws

## Discussions

In the existing study, researchers investigated the status of tobacco consumption and compliance with TCLs in all institutional tires of NUB. According to the Global Adult Tobacco Survey Report 2017, the basic prevalence of tobacco smoking in Bangladesh was found 18% among which the majority (53.2%) had initiation of smoking during their teenage,^18^ suggesting a greater occurrence of smoking among college and university students in Bangladesh among younger adults in general. The prevalence among male university students in Bangladesh^19^ was found greater (68%) than that suggested in close nations such as India (20.4%),^20^ Pakistan (26.1%),^21^ Nepal (33.6%)^22^ and Malaysia (42.1%).^23^ It indicates that Bangladesh has a greater burden of tobacco rather than the neighbouring countries.

In this study, respondents from the younger age group (≤21 years: 78.4%, n=40/51) were observed to have substantial (p=0.01) non-satisfactory compliance with TCLs. Besides these, it was once shocking to see that college students from first year suggested most (75.8%, n=69/385) non-satisfactory compliance degree to the legal guidelines which may be a result of much less publicity to the tutorial platform and tobacco management activities. Another study in Bangladesh showed an increased risk of smoking among the third year students of Law faculty with positive attitudes towards tobacco-controlling measures among non-smokers.^24^ The qualitative approach of this study revealed a similar scenario that, senior students from law, business and IT departments tend to smoke more. However, the quantitative approach of this study showed more non-compliance with TCLs among the junior group.

Furthermore, it was once observed that positive family history (69.3%, n=52/385) and passive smoking status (64.3%, n=110/385) act as influencing triggers for non-compliance reputation towards TCLs (p=0.01). Moving towards standard know-how about TCLs, the study result indicates that the highest number of respondents had poor knowledge (94.8%, n=365/384) with a non-satisfactory compliance rate (57.8%, n=211/384). This outcome is supported by the study conducted among the male students of Rajshahi University in Bangladesh. For instance, it was revealed that students’ cigarette smoking behaviour is significantly triggered by the passive smoking status in family, parents and peers.^9 25^ Family and peer smoking status as predictors for the smoking behaviour of the students is revealed by many other researchers^726^,^28^ Another study conducted among Bangladeshi police revealed poor knowledge among them regarding various rules and clauses of NTCLs.^29^ A similar scenario revealed among the university students through this study indicates the neglected condition of tobacco control policy and its implications in Bangladesh which is alarming.

In addition, this study revealed significant non-satisfactory compliance among the students who initiated tobacco smoking during their teenage (9–19 years: COR/p=2.79/0.03; 95% CI 1.13 to 10.41) and had moderate levels of nicotine dependence moderate nicotine dependency (AOR/p=4.04/0.01; 95% CI 1.28 to 12.74). This finding is supported by studies conducted among students of Bangladesh and India, where the mean tobacco initiation age of the smoker students was observed as 10.8 years and 17.3 years among whom Indians had mostly low nicotine dependency.^30 31^ That means rather than dependency peer influence played a vital role in the non-compliance behaviour of the students in academic institutions which might be mitigated by the implementation of strict policies of respective organisations. The Indian study was robust with a similar solution for such an issue.^31^

Regarding barriers and ways outs to reduce tobacco consumption, the study revealed that 127 out of 385 respondents who opined that their peers can’t influence them to quit tobacco, had very low compliance on TCLs as they were stubborn and passionate about tobacco smoking. However, in searching for a way out to minimise tobacco consumption, it was discovered that respondents who recommended family guidance (69.1%, n=47/385) and teacher support (52.4%, n=130/385) had a significant non-satisfactory compliance with TCLs at university setting. In another study, family guidance was suggested by the researchers from the outcome of the study conducted at Rajshahi University Bangladesh where the compliance status was not explored considering the TCLs.^25^ On the other hand, teachers’ smoking behaviour was identified as a significant predictor for the tobacco smoking status of the students.^30^ Therefore, a strict tobacco control policy is essential in the university setting which is suggested by the teachers of NUB in the qualitative survey of this study. Similar recommendations were found by other researchers.^7 25 31^ However, no study explored the opinions on the barriers and way-outs to minimise tobacco consumption in a university setting.

This study revealed a crucial scenario of TCLs implementation in the university setting though data were collected by telephonic interview during the COVID-19 lockdown period and direct observation of premises was not possible. In line with this limitation, this study took data from only one university which may affect the generalisability throughout Bangladesh. The unique exploration of compliance level on TCLs in a university setting is the strength of this study which might be a model for other studies to explore individual institutions’ compliance on TCLs. Moreover, this mixed-method exploration provided stronger evidence in this study. The outcome of this study might increase the awareness of reducing tobacco consumption among the university authorities in Bangladesh.

## Conclusions

This study explored an overview of the compliance status with TCLs in all tires of a university setting in a unique manner in depth. The outcome of this astute exploration focusing the university setup which is much more important to minimise tobacco consumption at the national level considering the cluster of similar types of institutions. The current study revealed a depressive scenario of compliance status on TCLs in all segments of the selected university setting. Non-satisfactory compliance was found in more than half of the cases. Similarly, the qualitative approach showed a worse illustration in this issue. Although tobacco is completely prohibited in the university area, compliance status was found very poor in this mixed-method approach. Non-satisfactory compliance is revealed significant among the younger aged first-semester group who got early initiation of smoking, had moderate nicotine dependency with poor knowledge of tobacco, did not agree with the barrier ‘peer group influence’ to combat tobacco and suggested family guidance as a possible sustainable way out to minimise tobacco consumption. A large-scale empirical study can be conducted by future researchers considering all the universities throughout the country to yield more valid and representative outcomes. The gaps regarding compliance with TCLs identified through this study will guide the government and policy-makers to plan for effective strategies and interventions through implementation programmes to keep the universities tobacco-free.

## supplementary material

10.1136/bmjph-2023-000496 online supplemental file 1

10.1136/bmjph-2023-000496 online supplemental file 2

## Data Availability

Data are available on reasonable request.
