# Supplementary material for: Compliance status of tobacco control laws in the university setting of Bangladesh: an analytical study followed a mixed-method approach
Source: BMJ Public Health. 2024 May 2;2(1):e000496. doi: 10.1136/bmjph-2023-000496 (PMC11812804; doi:10.1136/bmjph-2023-000496)
Supplement: online supplemental file 1 [file bmjph-2-1-s001.pdf]

Information Form

Study Title:

Opinions to minimize tobacco consumption and complying tobacco control law: setting approach in private university

Name of principal investigator: Dr. Nasrin Akter, Lecturer, Department of Public Health, Northern University Bangladesh.

Tobacco consumption is one of the major resistible cause for non-communicable diseases and an obstinate public health problem that charges threat to the health of the entire population. In the current pandemic situation of COVID-19, it has come to the concern that smoked tobacco consumers are badly suffering from COVID-19 and in most cases, it leads to death. Therefore, prevention of tobacco consumption can establish a sustainable wellbeing to combat all the curse and challenges regarding human health.

We are trying to identify the opinions to minimize tobacco consumption and to comply with tobacco control law among the population of Northern University Bangladesh through this study by which tobacco consumption can be minimized in educational areas. University authorities and management teams may get the guidance to minimize the tobacco use and enforcement of tobacco control law. The study will provide concrete information to plan awareness program to prevent tobacco consumption.

Since only an interview will take place, there is no risk in this research. You will not have any direct benefit for participating in this research but your participation will be helpful to create community awareness and significantly contribute to the development of long-term sustainable program, strategies and action plans that will contribute to minimize tobacco consumption and proper sustainable implementation tobacco control law as well as effective compliance of the legislation. There will be no physical, mental, economic & social harm to you for participation in this research.

Information provided by you will remain confidential. We shall not disclose this information to any other except the ethical committee and my supervisor. Your name or personal identity will not be published anywhere. Privacy will be maintained very strictly.

Right to accept or reject and withdraw your participation from the study:

Participation in this research is completely voluntary. If you desire, you may refrain from participating in this research. If you take part, you have the right to withdraw yourself at any time and any stage of the study.

Contact information

If you have any questions, you may ask now. If you want to know more during research, you may contact me:

Dr. Nasrin Akter
Lecturer,
Department of Public Health,
Northern University Bangladesh.
Phone: 8801675746599.

Consent for participating in this research

I am invited to participate in the research titled “Opinions to minimize tobacco consumption and complying tobacco control law: setting approach in private university”. Aim of the research, why I am invited to participate, expectation from me, risk and benefit of the research is informed to me in details.

I have got satisfactory answer to my questions related to the research. I will fully give consent to participate in this research and I know that, any time, despite the consent, I may withdraw myself from the research

……………………… ………………..……..… ………………

Name of the participant Signature of the participant/ Date
thumb impression

…………………………… ……………..

Signature of the researcher
Date

English Questionnaire

Section 1: Survey Information			
**Sl No**	**Question**	**Response** (*Encircle appropriate response*)	**Code** (*Write code no.*)
1.01	Interview ID		I__I I__I__I__I 1.01
1.02	Name	………………………………………………………………………………… 1.02	
1.03	Department	a. Name of the Department	……………………………………………… 1.063a
1.04	Do you have any contact mobile number?	1 = Yes<br>2 = No ***If No, then go to the question no. 1.06***	I__I 1.04
1.05	Would please tell me your contact mobile number?		I___I___I___I___I___I___I___I___I___I ___I___I 1.05
1.06	*Interviewer: state here your name and signature Name……………………………………… ……………………………………………… ………Signature:……………………… …………*		I__I 1.10

Section 2: Socio-Demographic Information				
**Sl No**	**Variable**	**Question**	**Response** *(Encircle appropriate response)*	**Code** *(Write code no)*
2.01	Age	How old are you (*age of the patient in completed years*)		I__I__I 2.01
2.02	Sex	Sex ***(Please fill this question without asking)***	1 = Male	I__I 2.02
			2 = Female	
2.03	Education	What is the class/ level of education that you are studying now?	1= 1st Semester	I__I__I 2.03
			2= 2nd Semester	
			3= 3rd Semester	
			4= Final Semester	
2.04	Father's Education	What is your father's last education?	1. No formal education	I__I__I 2.04
			2. Upto Primary	
			3. Secondary	
			4. Higher Secondary	
			5. Graduation or above	
2.05	Mother's Education	What is your mother's last education?	1. No formal education	I__I__I 2.05
			2. Upto Primary	
			3. Secondary	
			4. Higher Secondary	
			5. Graduation or above	
2.06	Religion	What is your religion?	1 = Muslim	I__I 2.06
			2 = Hindu	
			3 = Christian	
			4 = Buddhist	
2.07	Income	Monthly family income		...........................2.07
2.08	Father's Occupation	Which of the following best (in terms of time) describes your father's work status over the last 12 months?	01 = Government employee	I__I__I 2.08
			02= Non-government employee	
			04=Self-employed/ Business	
			04=Farmer	
			05=Jobless	
			06=Student	
2.08a	Specify occupation		88=Others (specify)	...........................2.08a
2.09	Mother's Occupation	Which of the following best (in terms of time) describes your mother's work status over the last 12 months?	01 = Government employee	I__I__I 2.09
			02= Non-government employee	
			03=Self-employed/ Business	
			04=Housewife	
			05=Student	
2.09a	Specify occupation		88=Others (specify)	...........................2.09a

**Section 3: Tobacco consumption pattern among the students**				
**Sl No**	**Variables**	**Questions**	**Response** *(Encircle appropriate response)*	**Code** *(Write code no)*
3.01	Tobacco consumption status	Do you consume tobacco? **(If yes, please fill up 3.02)**	1= Yes	I__I 3.01
			2= No	
3.02	Types of consuming tobacco	If Yes, what is the type of consumed tobacco? **(If yes, fill up 3.03) Multiple**	1 = Cigarette	I__I 3.02
			2 = Bidi	
			3 = Hukkah	
			4 = Pipes	
			5 = Cigars/chiruts	
			6 = Smokeless tobacco (jarda/sadapata/gul/khoinee)	
			7 = Electronic cigarette	
3.03	Consumer type	If yes, What is your consumption status?	1 = Current consumer	I__I 3.03
			2 = Chronic consumer	
			3= Occasional	
			4 = Never consumed	
3.04	Duration of tobacco consumption	At what age you started consumption of tobacco?	……….. 3.04	
3.05		For how many years are you consuming tobacco?	……….. 3.05	
3.06	Dependency on smoked tobacco	How soon after you wake up do you smoke your first cigarette?	1. Within 5 minutes	…….. 3.06
			2. 31 to 60 minutes	

			3. 6 to 30 minutes	
			4. After 60 minutes	
3.07		Do you find it difficult to refrain from smoking in places where it is forbidden?	1 = Yes	I__I 3.07
			2 = No	
3.08		How many cigarettes per day do you smoke?	1. 10 or less	I__I 3.08
			2. 11 to 20	
			3. 21 to 30	
			4. 31 or more	
3.09		Do you smoke more frequently during the first hours after waking than during the rest of the day?	1. Yes	I__I 3.09
			2. No	
3.10.		Do you smoke when you are so ill that you are in bed most of the day?	1 = Yes	I__I 3.10
			2 = No	
3.11		Do you want to quit tobacco?	1= Yes	I__I 3.11
			2= No	
3.12	Passive smoking status	Passive smoking at home?	1. Yes	I__I 3.12
			2. No	
3.13		Father smoking	1. Yes	I__I 3.13
			2. No	
3.14		Mother smoking	1. Yes	I__I 3.14
			2. No	
3.15		Other family member smoking	1. Yes	I__I 3.15

			2. No	
3.16		Peer group (Friends, teachers at university) smoking	1. Yes	I__I 3.16
			2. No	
3.17	Barriers to minimize tobacco consumption	Which factor can be the barrier for minimizing or quitting tobacco consumption from your perspective?	1. Negative self willingness	I__I 3.17
			2. Influence from peer group (friends/relative/teachers etc)	
			3. Influence from social media	
			4. Do not having sufficient knowledge on Tobacco Control Law	

**Section 4: Knowledge regarding health hazards of tobacco intake and compliances with tobacco control law**				
**Sl No**	**Variables**	**Questions**	**Response** *(Encircle appropriate response)*	**Code** *(Write code no)*
4.01	Knowledge on hazards of Tobacco consumption	Do you know about the health hazards associated with smoking/smokeless tobacco?	1= Yes	I__I 4.01
			2= No	
4.02		If yes, what type of health hazards? (Multiple response)	1 = Lung cancer	I__I 4.02
			2 = Asthma	
			3= Cardiovascular diseases	
			4= loss of memory	
			5= Throat Cancer	
			6= All of above	
4.03		Do you know the nicotinic effect of secondary exposure of smoking on family?	1= Yes	I__I 4.03
			2= No	
4.04		Do you know the nicotinic effect of secondary exposure of smoking on children?	1= Yes	I__I 4.04
			2= No	
4.05		Do you know the tobacco control laws?	1 = Yes	I__I 4.05

	Knowledge on tobacco control law		2 = No	
4.06		If yes, what are the laws practicing under National Tobacco Control Law in Bangladesh?	1. Fines for non-compliance of smoke free rule in public place should be applied	I__I 4.06
			2. Anti-tobacco messages will be shown if tobacco use is included in a movie	
			3. Sales of tobacco to and by minors have been banned	
			4. Graphical health warnings are to be printed on tobacco packs	
			5. Any form of tobacco advertisement is prohibited in any selling products, if anyone contravens he shall be punished with simple imprisonment	
			6. Smoking is prohibited in the majority of indoor public places and workplaces, healthcare facilities and educational institution	
			7. Specified places for smoking should be marked off by the ownar of the public place or vehicle.	
			8. "Be abstain from smoking, it is a punishable offence" this vigilance notice should be exibited in public area and vehiclec by the ownar/manager/caretaker of the area.	

4.07		Do you know smoking or tobacco consumption is prohibited at indoor and outdoor University area?	1 = Yes<br>2 = No	I__I 4.07
4.08		Which one is the fine for non-compliance of tobacco control law regarding smoking in public place? (BDT)	1. 300<br>2. Don't know	I__I 4.08
4.09		How much area of the cigarette packet should be covered by graphical health warning?	1. 50%<br>2. Don't Know	I__I 4.09
4.10		Do you know the steps of quiting tobacco?	1= Yes<br>2= No	I__I 4.10
4.11		If yes what are the steps to quit tobacco?	1 = Set a quit date within 2 weeks<br>2 = Take support from family and friends<br>3 = Identify past experiences. What worked? What didn't<br>4 = Anticipate challenges of Symptoms<br>5 = Remove tobacco products<br>6 = Ask tobacco user family members, not to smoke around patient or leave their tobacco products where he/she can get them during their quit attempt<br>7 = Avoid betel leaf<br>8 = Avoid alcohol<br>9= Willingness to quit tobacco products	I__I 4.11

			88 = Others (specify)	
4.12	Compliance on tobacco control law	Have you ever been smoked tobacco inside University area? (Consumers Only)	1. Yes<br>2. No<br>3. Not applicable	I__I 4.12
4.13		Have you ever been smoked tobacco outside University area at public place?	1. Yes<br>2. No<br>3. Not applicable	I__I 4.13
4.14		Do you prohibit friends/peers to smoke in public places?	1. Yes<br>2. No	I__I 4.14
4.15	Status of implications of tobacco control laws in the University areas	Does your teacher advice you to avoid or minimise tobacco consumption?	1 = Yes<br>2 = No	I__I 4.15
4.16		Did you see any anti tobacco advertisement in your University area? (Indoor and outdoor)	1 = Yes<br>2 = No	I__I 4.16
4.17		Do you ever see selling or buying tobacco (cigarette) by person under 18? in University area	1 = Yes<br>2 = No	I__I 4.17
4.18		Did you ever see someone selling single cigarettes or any package with less than 20 cigarettes in university area or other public areas?	1 = Yes<br>2 = No	I__I 4.18
4.19		Ever seen any special marked areas for smoking zone in university area?	1= Yes<br>2= No	I__I 4.19
4.20.		Did you ever seen no smoking sign in University zone?	1= Yes<br>2= No	I__I 4.20
4.21		Did you ever seen no smoking sign in	1= Yes	I__I 4.21

		University cafeteria or restaurant?	2= No	
4.22		Ever seen no smoking sign in public bus or university bus?	1= Yes	I__I 4.22
			2= No	
4.23	Opinion on minimizing tobacco	How do you feel to quit tobacco? You have to say (Consumer only)	1. Very Interested	I__I 4.23
			2. Interested	
			3. Neutral/ Confused	
			4. Not Interested	
			5. Strongly not interested	
4.24		Which one would be more helpful to minimize tobacco consumption among the university students?	1. Self influence	I__I 4.24
			2. Friend's influence	
			3. Family guidance	
			4. Teacher's support	
			5. Laws implimentations by the University	
