# Supplementary material for: Compliance status of tobacco control laws in the university setting of Bangladesh: an analytical study followed a mixed-method approach
Source: BMJ Public Health. 2024 May 2;2(1):e000496. doi: 10.1136/bmjph-2023-000496 (PMC11812804; doi:10.1136/bmjph-2023-000496)
Supplement: online supplemental file 2 [file bmjph-2-1-s002.pdf]

Key Informant Interview (KII) Guideline: English

Semi structured topic guide for (KII)

Respondent: Teachers of NUB

<u>Identification (ID) description</u>

1. **Individual Identification (IND ID):**
2. **Name:**
3. **Designation:**
4. **Department:**
5. **Phone number and Email ID:**
6. **Age:**
7. **Gender:**
8. **Education:**
9. **Work experience (in months):**

<u>Introduction:</u>

My name is ____________________ Lecturer, Department of Public Health, Northern University Bangladesh (NUB). We are trying to identify the opinions to minimize tobacco consumption according to national tobacco control laws in Bangladesh among the students, faculties and administrative staff of NUB through this study by which further development tobacco control law can be established, implemented and tobacco prevention can be sustainably facilitated in educational areas as well. I will ask you questions related to your knowledge & perception regarding tobacco consumption status, harmful effects of tobacco consumption, compliance with tobacco control law and tobacco prevention facilities in university area. Our conversation will be recorded by tape recorder and your information will be kept confidential, only data will be used analysis purpose. If you are agreed to participate in this survey, we need your verbal consent according to ethical consideration. Are you agree with me?

☐ Yes ☐ No

IND ID:

Setting description: (Dept)

Summary information:

Reflexive comments:

BACKGROUND INFORMATION ABOUT TOBACCO CONSUMPTION PATTERN AND STATUS IN UNIVERSITY CAMPUS:

1. Would you please tell me about recent tobacco consumption status in University area and how much do see that people within university consuming tobacco?

- Follow up points….
- Students (Which years are common?), Male, Female ratio
- Which Departments
- Your consumption status and other teachers (In your Department or another departments)

Note:

<u>RESPONDENT’S KNOWLEDGE REGARDING NATIONAL TOBACCO CONTROL LAWS IN BANGLADESH:</u>

2. If you know, would you please tell me about the tobacco control laws in Bangladesh?

- Yes
- No

- If yes, *interviewer will put tick mark below according to the response*
  - 300 Tk fine for non-compliance of smoke free rule in public place should be applied
  - Anti-tobacco messages will be shown if tobacco use is included in a movie
  - Sales of tobacco to and by minors have been banned
  - Graphical health warnings are to be printed on tobacco packs
  - Any form of tobacco advertisement is prohibited in any selling products, if anyone contravenes, he shall be punished with
  - Smoking is prohibited in the majority of indoor public places and workplaces, healthcare facilities and educational institution
  - Specified places for smoking should be marked off by the owner of the public place or vehicle.
  - "Be abstain from smoking, it is a punishable offence" this vigilance notice should be exhibited in public area and vehicle by the owner/manager/caretaker of the area.

<u>Note:</u>

RESPONDENT'S COMPLIANCE ON TOBACCO CONTROL LAWS WITH OPINIONS ABOUT IMPLEMENTATIONS IN THE UNIVERSITY:

3. Do you have any existing tobacco control laws or any policies to comply national tobacco control laws in in University?

- Follow up points….
- What are the laws
- What are the rules to implement the laws properly?
- How they are implemented? / how better implementations can be done in future?
- What are the policies
- Any other existing strategies

Note:

4. Have you ever been advised your students not to consume tobacco and counsel them against tobacco?

- Follow up points….
- about the health hazards of tobacco consumption
- informed students about the tobacco control laws
- any other information

Note:

5. Did you take any actions to minimize tobacco consumption in University area?

- Yes
- No

If yes, please explain….

<u>Note:</u>

6. Is there any policy to pay the fine due to tobacco consumption inside the University area?

- Yes
- No

If yes, please explain. (How much fine, who is taking and monitoring?)

<u>Note:</u>

7. Is there any policy to pay the fine due to tobacco consumption outside the University area within the campus? (Covered area….)

- Yes
- No

If yes, please explain.

<u>Note:</u>

8. Is there any policy to inhibit selling or buying tobacco products to the minors (below 18) outside the University but within campus? (Covered area for campus)

- Yes
- No

If yes, please explain……

Note:

9. Ever been arranged any anti-tobacco awareness program?

- Yes
- No

If yes, please explain. (which type and when arranged?)

Note:

10. Are there any existing policies to comply the tobacco control laws in University area?

- Yes
- No

If yes, please explain.

<u>Note:</u>

11. Did you see any specially marked smoking zone in university area?

- Yes
- No

If yes, please describe about the area and tobacco control policies.

<u>Note:</u>

12. Have you ever seen any poster of no smoking sign in university area?

- Yes
- No

If yes, please explain where did you see and how was that?

<u>Note:</u>

13. Have you ever seen any poster of no smoking sign in university cafeteria?

- Yes
- No

If yes, please explain.

Note:

14. Have you received any rules/advice to prohibit tobacco consumption in the University?

- Yes
- No

If Yes, please explain.

Note:

15. Have you ever consumed tobacco in University area? (If applicable)

- Yes
- No

Note:

RESPONDENT'S OPINION ON BARRIERS TO IMPLIMENT TOBACCO CONTROL LAWS IN THE UNIVERSITY AREA:

16. Have you ever found any barriers to implement or comply tobacco control laws in the University area? Would you explain me?

- Follow up points….
- How the barriers are acting?
- Ever they had been handled/ How they can be handled?

Note:

RESPONDENT'S OPINION ON THE WAYS TO OVERCOME THE BARRIERS TO IMPLIMENT TOBACCO CONTROL LAWS IN THE UNIVERSITY AREA:

17. Would you please share your opinion, about the strategies through which we can overcome the barriers to reduce tobacco consumption all over the University campus?

- Follow up points….
- What are the strategies?
- Whether there are any strategies can be followed especially for the students?
- Whether there are any strategies can be followed especially for the faculties?
- Whether there are any strategies can be followed especially for the administrative staffs?

Note:

18. Have you ever been found any student based functional or educational program at your University which was sponsored by any tobacco industry? / Did you found any program arranged by tobacco industry in your University?

- Follow up points…

- How do you feel it can be a barrier to minimize tobacco consumption in University area?
- How can overcome this barrier?

Note:

Exit questions

- Do you want to suggest anything regarding the punishment/fine to minimize tobacco consumption in university areas?

Note:

- Do you want to suggest anything regarding inhibition of tobacco advertisement/sponsorship by the tobacco industry in any program arranged by the universities?

Note:

- Do you want to suggest anything regarding the establishment or improvement of smoke free zone in the university area?

Note:
